# Towards Preventing Gaps in Health Care Systems through Smartphone Use: Analysis of ARKit for Accurate Measurement of Facial Distances in Different Angles

**DOI:** 10.3390/s23094486

**Published:** 2023-05-05

**Authors:** Leon Nissen, Julia Hübner, Jens Klinker, Maximilian Kapsecker, Alexander Leube, Max Schneckenburger, Stephan M. Jonas

**Affiliations:** 1 Institute for Digital Medicine, University Hospital Bonn, Venusberg-Campus 1, 53127 Bonn, Germanystephan.jonas@ukbonn.de (S.M.J.); 2 Carl Zeiss Vision GmbH, Turnstraße 27, 73430 Aalen, Germany; julia.huebner@zeiss.com (J.H.); alexander.leube@zeiss.com (A.L.); 3 School of Computation, Information and Technology, Technical University of Munich, Boltzmannstraße 3, 85748 Garching bei München, Germany; jens.klinker@tum.de; 4 Centre for Optical Technologies, Aalen University of Applied Science, 73430 Aalen, Germany; maxschneckenburger@web.de

**Keywords:** ARKit, face tracking, distance measurement, accuracy, vision assessment

## Abstract

There is a growing consensus in the global health community that the use of communication technologies will be an essential factor in ensuring universal health coverage of the world’s population. New technologies can only be used profitably if their accuracy is sufficient. Therefore, we explore the feasibility of using Apple’s ARKit technology to accurately measure the distance from the user’s eye to their smartphone screen. We developed an iOS application for measuring eyes-to-phone distances in various angles, using the built-in front-facing-camera and TrueDepth sensor. The actual position of the phone is precisely controlled and recorded, by fixing the head position and placing the phone in a robotic arm. Our results indicate that ARKit is capable of producing accurate measurements, with overall errors ranging between 0.88% and 9.07% from the actual distance, across various head positions. The accuracy of ARKit may be impacted by several factors such as head size, position, device model, and temperature. Our findings suggest that ARKit is a useful tool in the development of applications aimed at preventing eye damage caused by smartphone use.

## 1. Introduction

Digital technologies are shaping the future of global health and the Sustainable Development Agenda from the WHO highlights that the spread of information and communication technology has exciting potential to accelerate human progress [1].

However, global health care is challenged by the world’s growing and ageing population. In addition, the number of medical doctors per capita is decreasing in many regions [2,3]. For example, the availability of ophthalmologists varies widely by region and income level. The ratio of practising and trainee optometrists and ophthalmologists to population is only 3.7 per million in low-income countries, compared to 76.2 per million in high-income countries [2]. This highlights the need for targeted efforts to ensure that those affected by eye diseases can receive the care they need.

To address these challenges, there is a growing consensus in the global health community that the use of communication technologies will be an essential factor in ensuring universal health coverage for the world’s population [1].

The continuing evolution of smartphones, which are becoming faster, lighter, and equipped with more sensors [4,5,6], is expanding their areas of application and enhancing the accuracy and controllability of recorded data. Thus, not only digitization but also access to smartphones or mobile phones could prove critical in achieving the goal of universal health coverage.

Many people already track their daily lives with mobile devices such as smartphones, smart watches or similar [7]. For example, almost half of German population collect personal fitness, health or nutrition data or show an interest in doing so [8].

Measuring the distance between a mobile device and the user’s face can be a significant improvement for several health applications. For example, the utilization of automated distance measurements in the digitization of optometric tests, like an visual acuity check [9,10]. For such optometric tests, even small distance deviations can lead to a large influencing factor due to the triangular correlation of optotype size and the distance. To improve the accuracy for a camera-based distance measurement, a reference value can be used, e.g., the interpupillary distance [11] or a credit card (https://www.easee.online/, accessed on 3 April 2023). A wearable solution has been developed by Vivior (https://vivior.com accessed on 3 April 2023) to measure the distance to objects. The device is designed to be attached to glasses frames and features multiple sensors, including a depth sensor, for distance measurement. However, the sensor accuracy must be known in various application areas.

Another approach, developed by Vakunov et al. of Google Research, shows a method for measuring the distance between the smartphone and the eyes from an image, eliminating the need for an additional depth sensor by using the iris diameter as a reference value [12]. In addition, depth data can also provide enhanced information and replace reference values. A recent study by Taeger et al. has shown the potential of using facial recognition technologies such as Augmented Reality Kit (ARKit) and the TrueDepth sensor to assess facial motion disorders [13]. ARKit (https://developer.apple.com/augmented-reality/arkit/, accessed on 3 April 2023) is a framework developed by Apple Inc. for iOS devices that enables developers to create engaging and immersive augmented reality (AR) experiences. It uses the device camera and TrueDepth sensors to track and understand the environment, allowing digital content to blend seamlessly with the real world. With ARKit, users can interact with virtual objects and information overlaid onto the physical world, enhancing their overall experience. The TrueDepth sensor is a camera system used in Apple devices such as the iPhone X and newer models to create detailed 3D face maps for facial recognition features such as Face ID and to enable augmented reality applications. The TrueDepth sensor uses the vertical-cavity surface-emitting laser (VCSEL) technology to calculate the depth to an object [14]. It consists of a dot projector, an infrared camera, and a flood illuminator [14]. For the critical consideration of the power consumption, the use of other sensors, and the comparison of different device models as well as the measurement uncertainty, different iPhone models were tested.

The aim of this research was to explore the use of sensor data for digital health applications. The focus is on the accuracy of angle-dependent distance measurements taken with an iPhone X. In particular, the integrated ARKit framework and its ability to accurately measure distance to the user’s eyes using the integrated TrueDepth Sensor will be examined. For systematic results, data be collected using a custom-developed research app, measurements will be performed with an artificial head, and the various distances will be controlled and measured using an industrial robot. To demonstrate the applicability of the study to human heads, two additional measurements were conducted on individuals. This research will provide insight into the potential of using smartphones to improve the accuracy of smartphone-based distance measurements in digital healthcare, for example in optometric applications, and may influence the development of new digital health tools that can facilitate access to healthcare.

## 2. Material and Methods

To conduct a thorough analysis of the accuracy of the iPhone, several components were required. These include: a consistent naming system for all measurement positions, a custom-built application utilizing the iPhone’s sensor for data collection, a test setup, in which a robot scans an artificial head from different positions, and a methodology for analyzing the collected data.

### 2.1. Definition of Position

**Figure 1:**
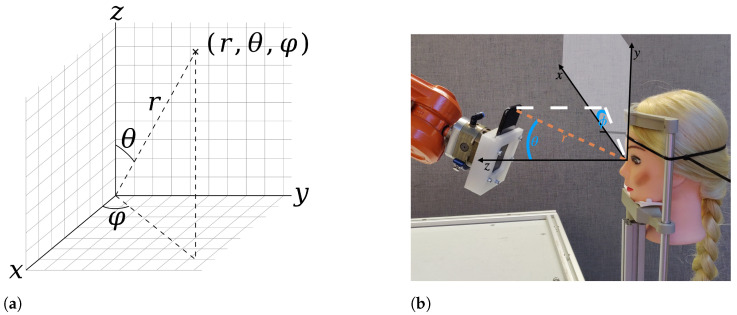
Description of the position. (**a**) Formal description of the coordinate system and positioning. (**b**) Real world setting with smartphone position in the upper right (UR) sight field and distance r=200 mm.

A Cartesian coordinate system (see Figure 1a) describes the relative position of the smartphone’s front speaker towards the head. The orientation of the smartphone to the face is managed by the robot (see Section 2.3) and always aligned to the center between both eyes. The head is placed in the center of the coordinate system, i.e., at position (0,0,0). The straight viewing direction corresponds to the z-axis. A robot arm moves the smartphone around the head (see Figure 1b). The unique position of the smartphone in relation to the head is then given by spherical coordinates. That is the triple $P_{r,\theta ,\varphi }=\left(r,\theta ,\varphi \right)$ in which the value *r* is the distance between head and smartphone given in the measuring unit mm. θ and φ are the angles in degree to describe the rotation in the x−y plane and along the polar axis, respectively. Thus, if the smartphone is placed frontal to the head with a distance of 200 mm, the coordinates (200,0,0) do represent the position. For the purpose of readability, Table A1 introduces a reference on the predefined measuring positions and allows the usage of abbreviations. E.g., the fixed angle pair $P_{r,11.25^{\circ },-11.25^{\circ }}=\left(r,11.25^{\circ },-11.25^{\circ }\right)$ is denoted by $P_{r,UL1}=\left(r,UL1\right)$ to indicate a position in the upper left sight field of the head for a non-specified distance *r*. In this experiment *r* is an element of R={200,250,300,350,400,450,500,550,600}. All data were collected from 200 mm to 600 mm, with 200 mm being the lower threshold as eye tracking is limited below this point. The upper threshold was determined based on the average arm reach per DIN 33402-2.

### 2.2. Smartphone Application

A case-study approach was used which comprised a statistical analysis of the smartphone sensors’ accuracy. The research was conducted using the 2017 Apple iPhone X (Apple Inc., Cupertino, CA, USA) as the primary smartphone. The iPhone X features an A11 Bionic Chip, 5.8-inch display, and 7-megapixel front-facing camera with the first version of the TrueDepth sensor for facial recognition and depth sensing.

We developed an iOS application to analyze the accuracy and investigate the capabilities of the TrueDepth sensor. The app was developed using Apple’s Integrated Development Environment (IDE), Xcode, and programmed it in Swift to create a native iOS application that utilizes the ARKit framework. ARKit is a framework developed by Apple to integrate augmented reality experience into apps or games. Currently, ARKit can add 2D or 3D elements into the live view from the device’s camera and also keep track of other objects in the scene. Our application was developed using ARKit 4, which was released by Apple in September 2020. ARKit can also be used to analyze the user’s face. In our development, we selected the ARFaceTrackingConfiguration, which uses the front camera and the TrueDepth camera system. The TrueDepth camera system creates a depth map of the user’s face. ARKit utilizes the front camera’s RGB image and the TrueDepth sensor’s depth map to determine distances and facial landmarks. Our prototype app integrates ARKit and utilizes the ARSCNView and its delegate functions to access the ARFaceAnchor and properties for the left and right eyes (leftEyeTransform, rightEyeTransform). By using the null vector in ARKit’s coordinate system, which represents the camera, we can calculate the distance between the user’s eyes and the phone. The front camera of the iPhone X can record videos at 60 frames per second (FPS), enabling us to recalculate distances every 16.67 milliseconds. In general, the TrueDepth sensor is limited to 30 FPS, but with ARKit we get depth information at only 15 FPS. To ensure accurate distance measurements, we only calculated distances when both the RGB image and depth map were available. This approach aims to ensure that ARKit utilizes the most recent depth information, rather than relying on outdated data or the computer vision processing of the RGB image. As a result and to achieve a stable measurement, we saved data at frame rate of 10 FPS. Sensor data such as device motion, magnetometer data, and temperature level inside the iPhone were also included with each distance data point for further analysis. All data is saved on the iPhone’s storage and can be exported as needed.

### 2.3. Test Setup

To fully evaluate the capabilities of the TrueDepth sensor on the iPhone, we conducted three experiments. Firstly, we assessed the distance measurement accuracy. Secondly, we examined the energy consumption of the sensor. Lastly, we evaluated the measurement uncertainty and compared results across different iPhone generations.

#### 2.3.1. Distance Measurement

**Figure 2:**
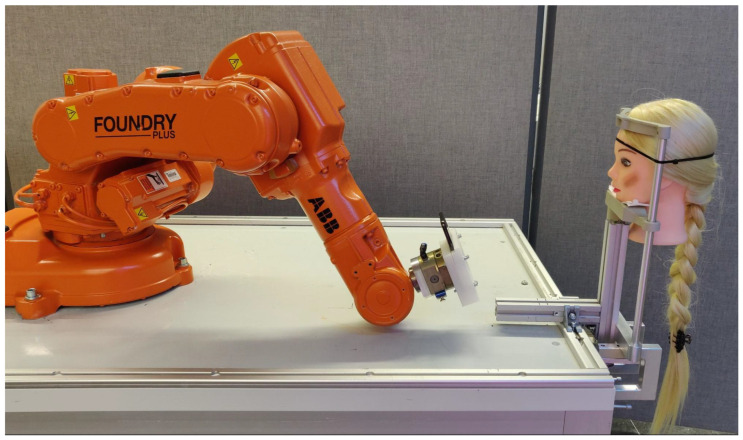
Test-setup including industrial robot, iPhone X on custom-made mounting bracket and the artificial head on the headrest.

Our experimental setup included an industrial robot, an iPhone X, a custom-made mounting bracket for the iPhone, a chin and headrest, an artificial head, and aTable (Figure 2). The use of an industrial robot enabled us to achieve precise and reproducible distance measurements of up to 0.03 mm. The table was used to mount the robot and other equipment. The robot we used for the measurements was an ABB IRB 140, which has six degrees of freedom and a payload capacity of 6 kg. A special mounting bracket was designed and 3D printed to precisely hold the iPhone on the robot. Our custom-made mount eliminated potential sources of error such as vibrations or smartphone misalignment caused by robot movement. Additionally, the mount allowed us to control the app, change settings and start and stop measurements while the iPhone was securely mounted on the robot. The exact dimensions of the mount ensured that we could position the smartphone with the robot precisely. To obtain accurate measurements on human subjects, we employed a standardized chin and headrest commonly used in optometry experiments. This eliminated a significant number of measurement inaccuracies caused by unexpected head movement. In order to have consistent lighting conditions, we used laboratory lighting conditions simulating daylight.

Tölgyessy et al. employed a comparable experimental arrangement, utilizing a robot to accurately move a plate and measure distance, as we did in our study [15].

In order to improve the precision of our measurements, we employed the use of an artificial head constructed of rubber that featured a circumference of 510 mm with an Interpupillary Distance (IPD) of 60 mm and incorporated naturalistic facial landmarks such as a mouth, eyes, and nose. It is noteworthy that the dimensions of the artificial head were 8.52% (circumference of 510 mm) smaller in comparison to the standard established in DIN 33402-2 (male: 570 mm, female: 545 mm). In DIN 33402-2 the German national organization for standardization defines several human body dimensions such as the head circumference. To provide a point of reference, we also conducted measurements using two human participants, specifically with head circumferences of 545 mm (female, IPD 64 mm) and 573 mm (male, IPD 66 mm). Having various head sizes enabled us to conduct a comparative analysis between the measurements obtained from the artificial head and those obtained from human subjects. To gain a complete understanding of the sensor’s accuracy, we measured the distance in various positions in front of the face. To achieve this, we implemented a strategy in which the robot moves around the head. We selected frontal, top, down, left, right and the diagonals with angles for θ and $\varphi =\{0^{\circ },\pm 11.25^{\circ },\pm 22.5^{\circ }\}$, respectively. However, due to the limited working space of the robot, we were not able to measure at all positions all r={200,250,...,600} for all pair of angles, resulting in 16 positions total (see Table A1). At the most positions, the robot moves between 200 mm and 600 mm away from the head in 50 mm intervals and stops for five seconds for the app to capture around 150 images. To eliminate data captured while the robot was moving, we synchronized the robot’s movement with the data captured by the app using the current time on both devices. For synchronization between the robot and the iPhone, we used accurate time measurement. The iPhone’s precision is in microseconds, while the robot’s precision is milliseconds. This allows us to synchronize movements with the measurement. For all positions the test run took approximately 15 min. Additionally, we measured the distance also while moving (1 mm/s) the smartphone continuously (r=[200,600]) at all positions, as described in Table A1. This approach has several advantages, including the ability to investigate the sensor’s accuracy during movement.

#### 2.3.2. Energy Consumption

Measuring distance using the iPhone’s camera and TrueDepth sensor consumes a significant amount of energy. To analyze this energy consumption, we conducted a separate experiment in which we added a feature to our prototype application that allowed us to control the frame rate of the distance measurement and record the corresponding battery level provided by the system. In our test setup, we selected to capture a distance measurement every 10 s. After the distance was calculated, we paused the ARKit session, which disabled the camera and TrueDepth sensor. However, in some cases, ARKit was unable to find the user’s face, causing the session to continue, which led to incorrect energy consumption. To address this issue, we set a timeout of one second after which the session would be paused even if no distance was calculated.

We monitored the battery level of the iPhone by reading the value of UIDevice.current. batteryLevel together with the distance measurement every 10 s. In addition, we conducted an idle measurement to get a baseline for the energy consumption, when none of the sensors are active. During this measurement, we only captured the battery level without using any other sensors, with the same implementation.

To avoid energy usage by the iPhone screen, we presented a black screen which disables all pixels on OLED displays to not consume any energy. All recorded data was saved to the iPhone’s internal storage so that it could be exported after charging.

The experiment utilized two brand new iPhone 12 Pro devices. This ensured that the battery health was at 100% or few battery cycle counts and had not been degraded due to prior usage. The iPhone 12 Pro had a battery cycle count of less than 10. The battery cycle count is an indicator of how many times the phone has been fully discharged and recharged to 100%. Furthermore, the iPhone 12 Pro is equipped with a highly efficient chip, which allows for future-proof analysis.

#### 2.3.3. Device Model Comparison and Measurement Uncertainty

The TrueDepth sensor is available in several generations which include different features for the user. In this experiment we conducted a comparison study using additional iPhones with different sensor generations, including the iPhone 11 Pro, iPhone 12 Pro, iPhone 13 Pro, and iPhone 14 Pro. Through this experiment, our aim is to establish the generalizability of the results to other device models and eliminate the possibility of a faulty sensor in our primary measuring device, the iPhone X. To improve efficiency, we employed a simpler distance measurement method, eliminating the use of a robot. We mounted the iPhone on a table and centered the artificial head on the main axis of the camera. To evaluate the sensor uncertainty of the data during the distance measurement, we performed measurements at $P_{200,C}$, $P_{400,C}$, and $P_{600,C}$ for a 15-min time frame with a frame rate of 2 FPS. The process was repeated with each iPhone. During the measurement, we also analyzed the reported thermal state (https://developer.apple.com/documentation/foundation/processinfo/thermalstate, accessed on 3 April 2023) of the iPhone which describes the overall system temperature in four categories (nominal, fair, serious, critical). By analyzing these values, we aimed to investigate whether the thermal state of the iPhone influences the measured distance and whether it can affect the accuracy of the measurement. This data helps to quantify the measurement errors of any potential sources and improve the interpretation of the measurements. Through this approach, we were able to make valid comparisons across generations of iPhones and gain valuable insights about the performance of different sensor generations.

### 2.4. Analysis

The data captured by the prototype application was in JSON (JavaScript Object Notation) format. The timestamps for synchronization between the iPhone and the robot are text files containing the start time (HH:mm:SS), the position, with the current distance, and the relative time at which the robot arrives and departs at the position. The iPhone’s time reference was automatically synchronized with the internet time server (time.apple.com). The robot’s time was not synchronized automatically and had to be synchronized manually to the iPhone’s time before testing. With both pieces of information, we synchronized the data acquisition of the iPhone with the movement of the robot and separate the valid data points for the measurement from the data points in which the robot moved to a different position. The large size of the dataset allows for several types of analysis. This approach allows for better organization and efficient processing of the large dataset and enables a more detailed and accurate analysis of the results.

The distance between the iPhone and the face was measured from the camera to the midpoint between the eyes. Additionally, ARKit calculates the distance starting at the camera and ending in the center of the eyeball (https://developer.apple.com/documentation/arkit/arfaceanchor/2968191-lefteyetransform, accessed on 3 April 2023). However, the real distance to the left and right eye differs based on the viewing angle. During the analysis, we factored in both values—the actual distances to the left and right eye and the distance to the center of the eyeball. To correct the distance from the iPhone to the left and right eye, we used trigonometric functions (see Section 2.1). We calculated the correct values for each eye by using (1) the distance from the camera to the center of the eyes, (2) the distance between the center of the eyes to the corresponding pupil, and (3) the angle to the face. This calculation resulted in two values for the left and right eye. Additionally, we corrected the measured data by subtracting 11.7 mm which is the radius to the center of the eyeball [16]. This is necessary because the actual distance is calculated from the smartphone’s front speaker to the midpoint between both eyes on the head. This approach provides more accurate measurements of the distance to each eye and the center of the eye, resulting in a more precise assessment of visual acuity.

#### 2.4.1. Radial Plots

**Figure 3:**
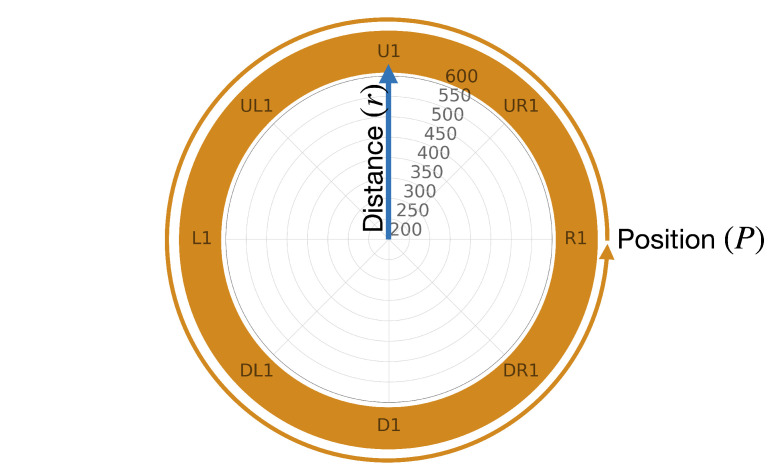
Description of radiation plot.

To accurately visualize the distance error in our study, we employed a radial plot and utilized a logarithmic scale with base *e* to ensure comparability across all distance measurements. The radial plot displays the positions (*P*), and the robots movement within the test, around the plot, as illustrated in Figure 3 (in orange color and direction for robot movements), and the distances (*r*) were added starting from the center of the plot and extending outwards towards the edges (blue arrow). We utilized a color scale ranging from green, representing lower distance error and greater accuracy, to red, representing higher distance error and reduced accuracy.

To determine the error, we computed the mean absolute error (MAE) in mm. As our data produced results below 1 mm, we added +1 to all values to avoid negative results (MAE+1), as values below 1 in the logarithmic scale (base *e*) are negative by definition. Hence we calculated the values as defined in Equation (3). Furthermore, we generated separate plots for angles of $\left(\theta ,\varphi \right)=\pm 11.25^{\circ }$ and $\left(\theta ,\varphi \right)=\pm 22.5^{\circ }$ to account for variations in measurement angles (see Section 2.1). It is worth noting that some positions in the diagram remain uncolored, as measurements were not feasible due to the limitations of the robot’s degrees of freedom.

(1) $$MAE=\frac{1}{n}\sum_{i=1}^{n}\left|x_{i}-y_{i}\right|$$

(2) $$MAPE=\frac{1}{n}\sum_{i=1}^{n}\left|\frac{x_{i}-y_{i}}{x_{i}}\right|$$

(3) $$\log_{e}\left(MAE\right)=\frac{1}{n}\sum_{i=1}^{n}\left(\right|x_{i}-y_{i}\left|+1\right)$$

## 3. Results

The results of our study can be divided into four sub-results. Section Distance Error shows the accuracy at different positions, while Section Reproducibility presents the consistency over multiple measurements. We also analyzed the Temperature and Sensor Uncertainty and the Energy Consumption to investigate potential issues in accurate measurements.

### 3.1. Distance Error

We separated the results into Central Position in which we analyzed only the positions central to the face. In Section Angle Positions we investigated various positions in front of the head. Lastly, in Section Human Measurements we analyzed the influence of larger human faces compared to the artificial head.

#### 3.1.1. Central Position

**Figure 4:**
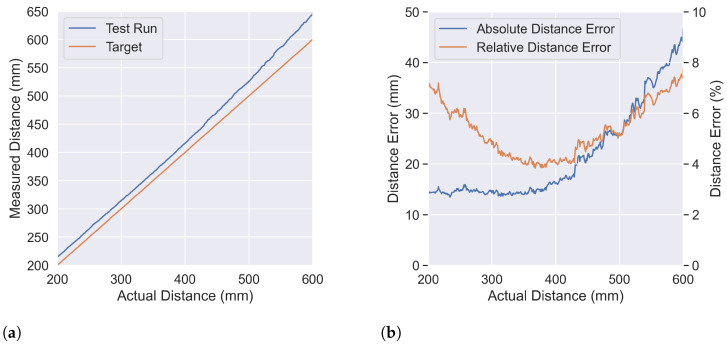
Distance measurement at $P_{R^{\prime },C}$ where $R^{\prime }$ is continuously defined ($R^{\prime }=\left[200,600\right]$) and the robot moves at a speed of 1 mm/s. (**a**) Measured distance (blue) vs. actual distance (orange) with zero error represented by the orange line. (**b**) Absolute (blue) and relative error (orange) in measured distance.

The first analysis focused on the position $P_{R^{\prime },C}$, where $R^{\prime }$ is continuously defined and the robot moves at a speed of 1 mm/s. Figure 4a illustrates the actual and measured distance in millimeters. The data show that until 400 mm, the measured distance is slightly higher than the actual distance. However, as the distance increases to 600 mm, the error between the measured and actual distance also increases consistently, with a discrepancy at the end of 45 mm. Figure 4b illustrates the absolute error in millimeters and percentage between the actual and measured distances. The plot shows a consistent error rate of 15 mm until a distance of 400 mm, after which the error rate increases. The lowest error rate of 3.8% is observed at an actual distance of 367 mm. Beyond this point, both above and below, the error rate increases, reaching a maximum of 7.5% at a distance of 596 mm. On average, the error rate across all distances between 200 mm and 600 mm is 5.32%. This data analysis highlights the error rate of one position at various distances, providing insight into the accuracy of the measurement.

#### 3.1.2. Angle Position

The percentage errors presented below have been calculated in accordance with the formula defined in Equation (2) in Section 2.4.1.

**Figure 5:**
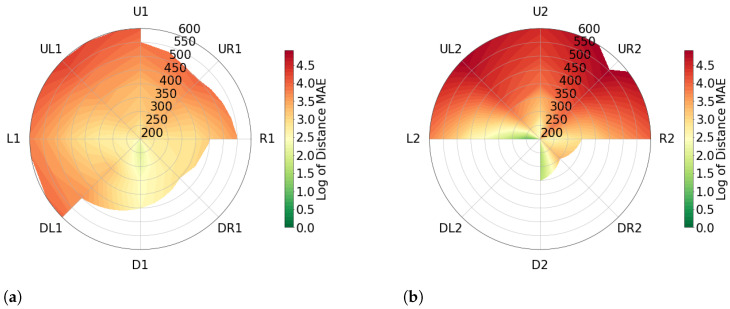
Error in angular positions for the artificial head visualized with mean absolute error on a logarithmic scale. Grid lines show measured values, interpolation between them. White areas indicate missing data. (**a**) represents (θ,φ)=±11.25 and (**b**) represents (θ,φ)=±22.5.

Figure 5 presents two plots for an artificial head. Figure 5a illustrates the distance error in at $\left(\theta ,\varphi \right)=\pm 11.25^{\circ }$ while Figure 5b represents the distance error of $\left(\theta ,\varphi \right)=\pm 22.5^{\circ }$. The average distance error for all positions measured at an angle of $\left(\theta ,\varphi \right)=\pm 11.25^{\circ }$ is 6.70%, while for positions measured at $\left(\theta ,\varphi \right)=\pm 22.5^{\circ }$ the average distance error is 10.36%. An interesting observation from Figure 5 is that the highest level of accuracy is seen in measurements taken at positions $P_{R,D1}$, with an average distance error of 2.89%. In contrast, the worst results are observed at the $P_{R,UL1}$ and $P_{R,UR1}$ positions, with average distance errors of 8.73% and 9.48% respectively, when measuring at the same angle.

Even though measurements taken at the position $P_{R^{\prime },D2}$ with limited $R^{\prime }\in \{200,250,$300,350}, the accuracy level is still high with an average distance error of 1.60%. However, positions $P_{R,UL2}$ and $P_{R,UR2}$ exhibit poor performance with average distance errors of 14.82% and 20.17% respectively.

#### 3.1.3. Human Measurements

As the artificial head used in Section 2.3.1 is 8.52% smaller than an adult head, we conducted the experiment with two participants; one male (M.S.) and one female (J.H.), both aged between 20 and 30 years, to account for variations in head size.

**Figure 6:**
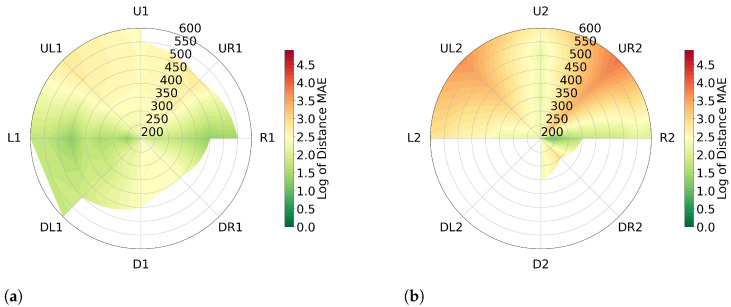
Error in angular positions for participant 1’s head (J.H.) visualized with mean absolute error on a logarithmic scale. Grid lines show measured values, interpolation between them. White areas indicate missing data. (**a**) represents (θ,φ)=±11.25 and (**b**) represents (θ,φ)=±22.5.

Figure 6 presents the plots for distance errors for both angles (θ,φ)=±11.25, and (θ,φ)=±22.5 in logarithmic scale for participant 1 (J.H., female). Figure 5 and Figure 6 can be compared as they use the same scale. Notably, the average distance error for all positions in both angles is significantly lower, at 2.61% and 3.03% respectively, in comparison to the measurements taken using the artificial head, which had average distance errors of 6.70% and 10.36%. A detailed examination of Figure 5b reveals a high level of error at the position $P_{R,UR2}$. Analysis of the data collected at this position shows that the error begins at the start of the measurement. The average distance error for this position is 8.11%, which is the worst performance across all positions. A similar level of error can be observed at the position $P_{R,UL2}$, with an average distance error of 5.25%. Both positions ($P_{R,UR2}$ and $P_{R,UL2}$) exhibit poor performance, as is also evident from the measurements taken using the artificial head.

**Figure 7:**
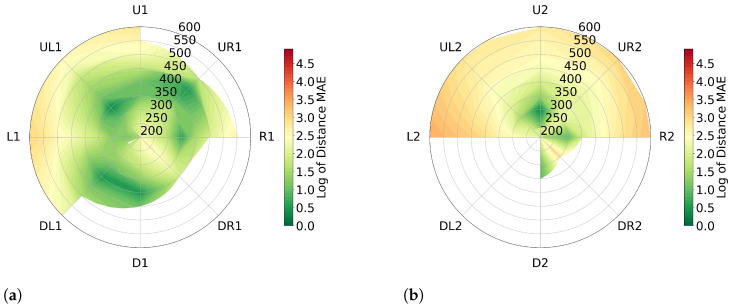
Error in angular positions for participant 2’s head (M.S.) visualized with mean absolute error on a logarithmic scale. Grid lines show measured values, interpolation between them. White areas indicate missing data. (**a**) represents (θ,φ)=±11.25 and (**b**) represents (θ,φ)=±22.5.

The data collected from the second participant (M.S., male) is presented in Figure 7. The overall distance error is 0.82% for measurements taken at (θ,φ)=±11.25 and 0.88% for measurements taken at (θ,φ)=±22.5. However, in comparison to the data collected from the first participant, it can be observed that similar levels of distance error are present at positions $P_{R,UL}$ and $P_{R,UL}$.

### 3.2. Reproducibility

#### 3.2.1. Distance

To analyze the consistency of the sensor, we conducted a different type of measurement. During this measurement, the robot moves along $P_{r,C}$ where r∈R. In order to analyze the consistency between results, we conducted the measurement multiple times under the same conditions in a short time interval. We quantified the reproducibility of the sensor’s results by computing the standard deviation of these measurements. Table 1 compares the measured distances from each experiment run and their corresponding reproducibility. The lowest standard deviation can be found at $P_{500,C}$, over all runs the difference between the measured distance is 0.64 mm. In contrast, at 350 mm, the standard deviation is the worst with 1.46 mm. On average, the standard deviation is 1.05 mm. This data provides information about the consistency of the sensor at different distances.

However, the values in Table 1 only represent the measurement directly in front of the head. Over all positions, all distances (*R*), and both angles ($\left(\theta ,\varphi \right)=\pm 11.25^{\circ }$ and $\left(\theta ,\varphi \right)=\pm 22.5^{\circ }$), the minimal, maximal and average standard deviation for 123 positions is 0.18 mm, 4.26 mm and 1.52 mm, respectively.

#### 3.2.2. Device Models

**Figure 8:**
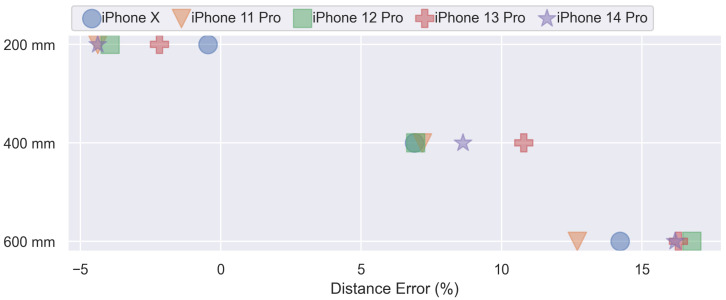
Average Error in Distance Measurement for iPhone Devices Across Various Models and Positions ($P_{200,C}$, $P_{400,C}$, $P_{600,C}$).

In our study, the primary device used to collect data was the iPhone X, which was utilized in all other experiments. To assess the consistency of results across different device models, an additional experiment was performed using five iPhone models (iPhone X, iPhone 11 Pro, iPhone 12 Pro, iPhone 13 Pro, and iPhone 14 Pro). It is important to note that in this experiment we used a simplified version of the test setup without the robot. The distance from $P_{200,C}$, $P_{400,C}$, and $P_{600,C}$ was recorded for multiple minutes using each device model. The results are presented in Figure 8 as the average error in percent. At $P_{200,C}$, the iPhone X shows a distance error of −0.46%, the iPhone 13 Pro exhibited an error of −2.18%, and the other models displayed an error of approximately −4.00%. At $P_{400,C}$, the iPhone X, iPhone 11 Pro, and iPhone 12 Pro displayed a distance error of 7%, while the iPhone 13 Pro and iPhone 14 Pro exhibited errors of 10.79% and 8.62%, respectively. At $P_{600,C}$, the iPhone X and iPhone 11 Pro displayed distinctive errors of 14.21% and 12.69%, respectively, while the other models displayed an error of approximately 16.00%. Additionally, it can be seen that at all positions, a few devices have similar error rates, with only two models standing out from that group. Finally, it can be noted that the error rate for all device models increases progressively with increasing distance.

### 3.3. Temperature and Sensor Uncertainty

**Figure 9:**
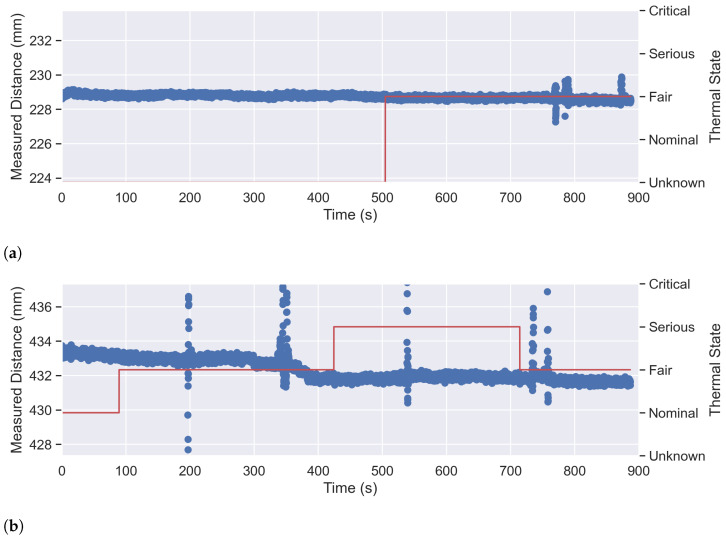

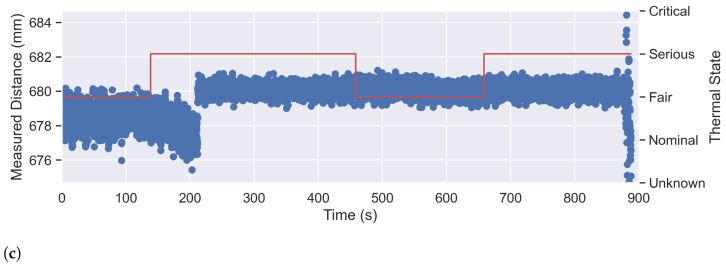
Plot of TrueDepth Sensor Uncertainty over Time at Positions at (**a**) $P_{200,C}$, (**b**) $P_{400,C}$, and (**c**) $P_{600,C}$ with the Current iPhone’s Thermal State (Red Line).

In the final phase of the experiment, we assessed performance over time by measuring the correct distance at three different distances ($P_{200,C}$, $P_{400,C}$, and $P_{600,C}$) for a period of 15 min. From previous measurements, we observed an increase in temperature on the iPhone’s display after a certain amount of time. To account for this temperature change, we also recorded the current temperature levels during the measurements. However, it should be noted that the iPhone’s operating system does not provide direct access to temperature in degrees Celsius and instead returns specific thermal states such as nominal, fair, serious, and critical. Figure 9 displays the static measurement results for the three different positions ($P_{200,C}$, $P_{400,C}$ and $P_{600,C}$). It’s important to note that the distance in this measurements are not corrected, since these plots should only represent the sensors measurement uncertainty and influence by temperature. The x-axis on the plots represents time in seconds, the left *y*-axis illustrates the measured distance, and the right *y*-axis displays the current thermal state. From Figure 9a ($P_{200,C}$), we can observe that the initial temperature level is unknown but increases to “Fair” after 10 min of measurement. After an additional 2 and a half minutes, the distance measurement becomes less accurate. Figure 9b ($P_{400,C}$) shows more variation in both the measured distance and the thermal state. The measurement starts with a thermal state of “Nominal”, but after 100 s the temperature level increases to “Fair”. After approximately 200 s, the plot reveals a noticeable presence of outliers. Similarly, at around 340 s, similar anomalies can be observed. However, immediately after, the values drop to a consistent distance of 432 mm. Notably, 20 s after this drop, the temperature level increases to “Serious”. Further on, after 520 s, we can observe a third spike. After 720 s, the iPhone’s thermal state reports a cooler temperature with the level of “Fair” again. Shortly after the cool down, we see another spike in the data, which, however, levels back to 432 mm after 20 s. At a distance of 600 mm $P_{600,C}$, Figure 9c displays a wider range of measured distances (3.44 mm) in the beginning. The temperature level started with “Fair” and increased to “Serious” after 140 s, followed by an outlier after 200 s. The remaining part of the measurement only shows a drop in temperature to “Fair” for about 200 s. However, during the cool-down, the measurement data does not show any significant outliers.

### 3.4. Energy Consumption

**Figure 10:**
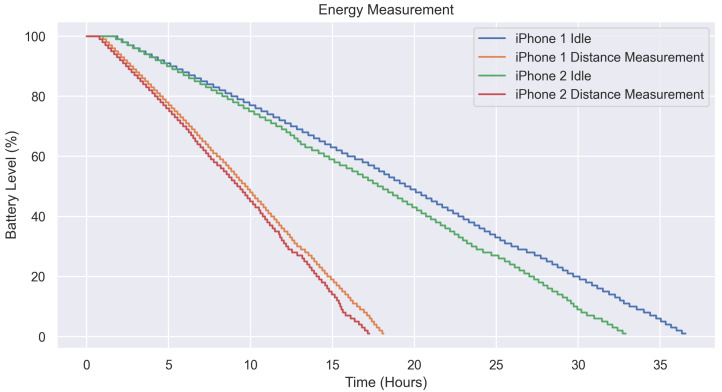
Comparison of Battery Level Over Time for Two iPhone 12 Pro Devices Running Prototype App with Distance Measurement (Every 10 s) Enabled and Disabled.

Figure 10 illustrates the energy consumption of the experiment outlined in Section 2.3.2. The plot displays the idle and distance measurement runs for both phones. iPhone 1 had a battery life of 36:32:59 (HH:MM:SS), while iPhone 2 had a battery life of 32:54:09, indicating a difference of 10% (03:38:49) in duration between the two phones. The duration of both iPhones when distance measurement was enabled was similar, at 18:07:09 for iPhone 1 and 17:15:19 for iPhone 2. This data demonstrates that enabling distance measurement significantly impacts energy consumption, reducing usage time by approximately 50%.

## 4. Discussion

An initial objective of the project was to determine if the iPhone’s TrueDepth sensor can be used in medical tests, such as during a vision assessment to improve test results. In this study, we conducted a baseline measurement in order to develop a broad understanding of how the sensor behaves and how accurate it is. Furthermore, we designed the study specifically to analyze the entire scope of the sensor so that the results can be used to develop various use cases.

### 4.1. Findings

In our study, we found strong evidence that the TrueDepth sensor’s accuracy ranges from 0.83% to 9.07% distance error. Specifically, measurements centered ($P_{R^{\prime },C}$) and below ($P_{R^{\prime },D}$) the head at a distance between 300 mm and 500 mm ($R^{\prime }=\left\{300,350,400\right\}$) are the positions with the highest accuracy. At some positions, limitations of the sensor and the experimental setup were encountered.

The finding of the lowest error rate (3.8%) at a distance of 387 mm, as reported in Section 3.2 of our study, raises important questions regarding the design and optimization of the sensor. One possible explanation for this result is that the sensor is optimized for distances within the range of 300–400 mm, which happens to be the average viewing distance of smartphones [17]. Moreover, our study revealed a higher error rate (7.35%) for closer distances, likely due to the camera’s inability to focus on objects in close proximity. Furthermore, we observed a gradual increase in error rate beyond 500 mm, which could be attributed to limitations in both the camera and the TrueDepth sensor’s resolution. These limitations may hinder the accurate detection of landmark positions over greater distances, as well as when the face is too close to the camera.

Although this theory is based on the workflow of ARKit, the software code cannot be directly investigated. The process is likely to involve (1) capturing an image of the user’s face, (2) locating the eye position, (3) matching the camera’s and the TrueDepth sensor’s coordinate systems, and finally (4) using the TrueDepth sensor to measure the distance to the eyes.

#### 4.1.1. Influence of Head Circumference

For our measurements, we chose to use the artificial head primarily for consistency, as humans are more prone to errors due to movements over the measurement period (approx. 15 min). Contrary to expectations, testing the same positions on actual human heads did not yield similar results. Participant 1 showed significantly better performance to the left ($P_{R,L}$) and right ($P_{R,R}$) of the head, rather than below. Participant 2, on the other hand, showed more accurate distances below the head ($P_{R,D}$). Additionally, an improvement can be observed for larger heads. For instance, the artificial head (head circumference of 510 mm) had an overall error of 6.70% at $\left(\theta ,\varphi \right)=\pm 11.25^{\circ }$ and 10.36% at $\left(\theta ,\varphi \right)=\pm 22.5^{\circ }$. Whereas participant 1 (head circumference of 545 mm) had an overall error of 2.61% at $\left(\theta ,\varphi \right)=\pm 11.25^{\circ }$ and 3.03% at $\left(\theta ,\varphi \right)=\pm 22.5^{\circ }$. Best results can be found at participant 2 (head circumference of 573 mm) with an overall error of 0.82% at $\left(\theta ,\varphi \right)=\pm 11.25^{\circ }$ and 0.88% at $\left(\theta ,\varphi \right)=\pm 22.5^{\circ }$. This indicates that there is a trend towards higher accuracy on larger head circumference. Furthermore, it’s possible that the observed effect may be confounded by the sex of the participants. Therefore, while these findings may provide some insight into the relationship between head size and accuracy, caution should be exercised in interpreting the results due to the small sample size.

#### 4.1.2. Continuous Error

An inconsistency we found in Participant 1’s measurement was a *ray of error* at the positions $P_{R,UL2}$ and $P_{R,UR2}$. It is difficult to explain this result, but it might be related to an error occurring at the beginning of this position measurement. The most likely explanation for this effect is that ARKit measured a wrong distance at the start, resulting from a previous measurement, and persisted with this error. To avoid this type of error, it may be helpful to restart the measurement each time the position changes. Doing so will prompt ARKit to recalibrate, which could potentially resolve the issue.

### 4.2. Influence of the Sensor Generation

The results of the experiment detailed in Section 3.2.2 indicate that our findings obtained using the iPhone X are consistent with other models and generations of the TrueDepth sensor. Moreover, it is observed that the error progressively increases as the distance increases. This trend in error can be addressed by implementing error correction functions, which can be developed through further data analysis. However, the discrepancy in the performance of newer models, such as the iPhone 14 Pro, which showed worse results compared to older models, can not be fully explained. One possible explaination for the difference can be that Apple has reduced the size of the TrueDepth camera system in the iPhone 13 Pro to fit into a smaller notch and the iPhone 14 Pro to fit into the dynamic island (https://support.apple.com/guide/iphone/view-activities-in-the-dynamic-island-iph28f50d10d/ios, accessed on 3 April 2023). It is also worth mentioning that Apple has enabled the use of the TrueDepth sensor for unlocking the iPhone even while wearing a mask on iPhone 12 models and later [18]. This demonstrates Apple’s improvement in accuracy by using the user’s eyes as the sole reference point for authentication. Expectations that new sensor generations would lead to better accuracy have not proved to be true. The reduced sensor size and different locations of the sensors be a possible explanation.

### 4.3. Influence of Temperature

The static measurements in Section 2.3.3 showed little influence of the smartphone’s temperature on the measured data. However, Apple advises developers to closely monitor temperature levels when using the TrueDepth sensor [19]. In our experiment we only saw temperature issues during test with the iPhone X. However, this data must be interpreted with caution because too little data was collected. Moreover, the measured data reveals a significant amount of noise, as well as several outliers. Notably, there is a discernible trend of increasing noise levels in correspondence to greater distances. It’s possible that this trend could be attributed to limitations in the resolution of the camera and TrueDepth sensor. Despite this, the data does not demonstrate a discernible pattern except for an escalated level of noise as the distance increases. In future investigations, it might be possible to collect more data over a longer period to analyze potential patterns and use more precise temperature measurement when conducting similar experiments.

### 4.4. Comparison with Literature

After conducting extensive research, we were unable to locate literature that specifically examines the measurement of the distance between a smartphone and a human face. Nevertheless, we did discover comparable studies that explore the precision of the TrueDepth sensor in measuring the distance to objects.

Breitbarth et al. conducted research on the accuracy of the TrueDepth sensor and found that errors can reach up to 5% of the target distance. Additionally, they discovered that stable measurements are achievable starting at a distance of 300 mm, with a minimum working distance of between 150 mm and 170 mm [20]. While these results are consistent with our own measurements, it is important to note that Breitbarth et al. used a different framework (AVFoundation) to access the TrueDepth sensor data and measured the distance to a specific point. In contrast, we utilized the ARKit framework to obtain the position of the eyes and compute the corresponding distance.

The TrueDepth sensor technology in the iPhone is similar to Microsoft’s distance-measuring sensor Kinect, as noted in Breitbarth et al.’s research. According to Tölgyessy et al. and Kurillo et al.’s studies, the newest version of the Kinect sensor, Azure Kinect, has high accuracy. Azure Kinect has an error rate of less than 11 mm [15] and exhibits the best accuracy in the range of 2500 mm to 3500 mm [21].

These findings demonstrate that depth sensors can be developed for specific use cases, and our results are similar in accuracy to their designated distance range.

### 4.5. Limitations

It is important to acknowledge some limitations encountered during the course of this research.

The limited range of motion of the robot which hinders us to measure at all distances for each position. This led to missing values at positions in which the smartphone is most commonly used.

Furthermore, synchronization between the measured distance by the phone and the robot’s movement presents a limitation in our experiment. We relied on the accurate time setting of both devices, but encountered difficulties in synchronizing them. The reliance on time led to imprecise synchronization compared to the overall setup, as some manual synchronization had to be done.

Measuring the distance to the user’s eyes with ARKit is straightforward, but the underlying processing of the image, the TrueDepth sensor data, and the internal computations, e.g., related to positioning in the middle eyeball, are not publicly documented and thus cannot be analyzed. Using ARKit’s face recognition to precisely locate the eyes in combination with the TrueDepth sensor data alone shows promising results and has the potential to improve the accuracy of distance measurements.

Finally, the relatively small amount of measurement data collected from human subjects is a limitation of this study. The lack of data on factors such as skin tone, eye color, head size, glasses, and other potential sources of measurement error may have limited our findings.

### 4.6. Future Work

Future work should make use of a more precise synchronization of the robot and the iPhone, techniques such as LabStreamingLayer (https://github.com/sccn/labstreaminglayer accessed on 3 April 2023) or a similar solution should be employed for synchronization.

Additionally, the accuracy of the distance measurement, particularly in the central and lower regions of the head, as well as within the range of 300–400 mm. Additionally, it would be beneficial to examine the gradual increase in error rates beyond 500 mm.

Finally, further research with more diverse human subjects is necessary to analyze facial features such as skin tone and eye color, head size, etc. and temperature effects on the TrueDepth sensor.

## 5. Conclusions

This study has demonstrated that the distance measurement using the iPhone’s TrueDepth camera system is generally accurate. The results suggest that the sensor is optimized for common smartphone usage scenarios, such as at a distance of 300 mm to 400 mm below the head. Applications requiring precise distance measurement can benefit from using the sensor in conjunction with the camera. However, the study also revealed that measurement errors are higher when the distance is above the head. Additionally, the distance measurement on human faces was found to be more precise than on the artificial head, potentially highlighting challenges in measuring smaller faces. The main limitation of this study was the discrepancy in size between the artificial head and human head, as well as the limited range of motion of the robot. In conclusion, the utilization of ARKit for measuring the distance to the user’s eye can have a range of applications in both active vision assessments and monitoring evaluations. 

## Figures and Tables

**Table 1:** Distance Measurements at Position ($P_{R,C}$) Over Five Tests with Resulting Reproducibility (Standard Deviation).

Position Name	Run 1	Run 2	Run 3	Run 4	Run 5	Standard Deviation (mm)
$P_{200,C}$	209.64	212.46	212.96	212.54	212.18	1.32
$P_{250,C}$	261.45	264.04	262.53	264.38	262.96	1.18
$P_{300,C}$	312.85	314.92	314.86	315.97	314.29	1.13
$P_{350,C}$	362.98	366.86	365.80	365.71	365.96	1.46
$P_{400,C}$	414.20	415.85	414.66	415.27	413.53	0.90
$P_{450,C}$	467.09	468.80	467.65	468.61	466.71	0.91
$P_{500,C}$	522.92	523.76	523.13	524.27	522.69	0.64
$P_{550,C}$	579.97	580.68	582.16	580.56	581.22	0.82
$P_{600,C}$	638.57	640.61	639.97	639.04	641.13	1.06

## Data Availability

The data supporting the findings of this study are available upon reasonable request. Please contact the corresponding author for access to the data.

## Abbreviations

The following abbreviations are used in this manuscript:

ARKit	Augmented Reality Framework
	developed by Apple Inc.
Face ID	Facial Recognition System
	developed by Apple Inc.
FPS	Frames per Second
RGB	Red, Green and Blue
MAE	Mean Absolute Error
JSON	JavaScript Object Notation

## Appendix A

**Table A1:** Predefined measuring positions of the smartphone.

Full Name	Short Name	θ-Angle	φ-Angle	Min *r* (mm)	Max *r* (mm)
Central	$P_{r,C}$	0	0	200	600
Up-1	$P_{r,U1}$	11.25	0	200	600
Up-2	$P_{r,U2}$	11.25	0	200	600
Up/Left-1	$P_{r,UL1}$	11.25	−11.25	200	600
Up/Left-2	$P_{r,UL2}$	22.5	−22.5	200	600
Left-1	$P_{r,L1}$	0	−11.25	200	600
Left-2	$P_{r,L2}$	0	−11.25	200	600
Down/Left-1	$P_{r,DL1}$	−11.25	−11.25	200	600
Down-1	$P_{r,D1}$	−11.25	0	200	450
Down-2	$P_{r,D2}$	−22.5	0	200	350
Down/Right-1	$P_{r,DR1}$	−11.25	11.25	200	400
Down/Right-2	$P_{r,DR2}$	−22.5	22.5	200	300
Right-1	$P_{r,R1}$	0	11.25	200	550
Right-2	$P_{r,R2}$	0	22.5	200	600
Up/Right-1	$P_{r,UR1}$	11.25	11.25	200	500
Up/Right-2	$P_{r,UR2}$	22.5	22.5	200	600
